# Chloroplast genome characteristics and phylogenetic analysis of *Scrophularia marilandica* Linnaeus (Scrophulariaceae)

**DOI:** 10.1080/23802359.2025.2555458

**Published:** 2025-09-03

**Authors:** Huiying Huang, Shuxin Du, Lei Guo, Mengli Zhou, Xinzhu Zhao, Fanhao Cui, Ruihong Wang

**Affiliations:** College of Life Sciences and Medicine, Zhejiang Sci-Tech University, Hangzhou, China

**Keywords:** Scrophularia marilandica, scrophulariaceae, chloroplast genome, phylogenetic analysis

## Abstract

*Scrophularia marilandica* Linnaeus 1753 is a herbaceous perennial medicinal plant of the family Scrophulariaceae, native throughout eastern and central North America. In this study, the first complete chloroplast genome of *S. marilandica* was reported and phylogenetic analysis was conducted with other 11 species from Scrophulariaceae. The chloroplast genome was 152,414 bp with 132 genes and includes a large single-copy (LSC) region (83,583 bp), a small single-copy (SSC) region (17,925 bp), and a pair of inverted repeat (IRs) regions (25,453 bp). The chloroplast genome reported here will be beneficial for its interspecific identification and evolutionary studies of *Scrophularia.*

## Introduction

The genus *Scrophularia* L., comprises more than 250 species mostly found across the Northern temperate zone, with many being used medicinally for eliminating heat and detoxifying (Hong 1983; Fischer 2002; Lee et al. 2021). *Scrophularia marilandica* L. 1753 a native plant of eastern and central North America, is commonly found along the edges of forests, by the sides of streams, and in lowland woodlands, and it is capable of thriving even in full sunlight (Brownstein et al. 2016). The root of *S. marilandica* can be made into tea, which is used to treat diseases such as fever and hemorrhoids, including inflammations (Hough 1849; Herrick 1977; USDA ARS 1992; Moerman 1998). It is also used as a diuretic and tonic, and can treat insomnia and anxiety as well. Previous studies have revealed the cell signaling pathways related to *S. marilandica* (Brownstein et al. 2021), laying a foundation for the research on its pharmacological mechanism of action. However, there is currently almost no research on the genomics of *S. marilandica*, which restricts the molecular mechanism study of the discovered pharmacological effects and its precise application in the medical field. In this research, we sequenced the chloroplast (cp) genome of *S. marilandica* for the first time, employing next-generation sequencing technology. Our primary emphasis was on examining the characteristics of the cp genome, identifying repeat sequences, and reconstructing the phylogeny. This investigation not only provides significant cp genome information but also enhances our comprehension of the evolutionary relationships among species within the Scrophulariaceae family.

## Materials and methods

**Figure 1.**
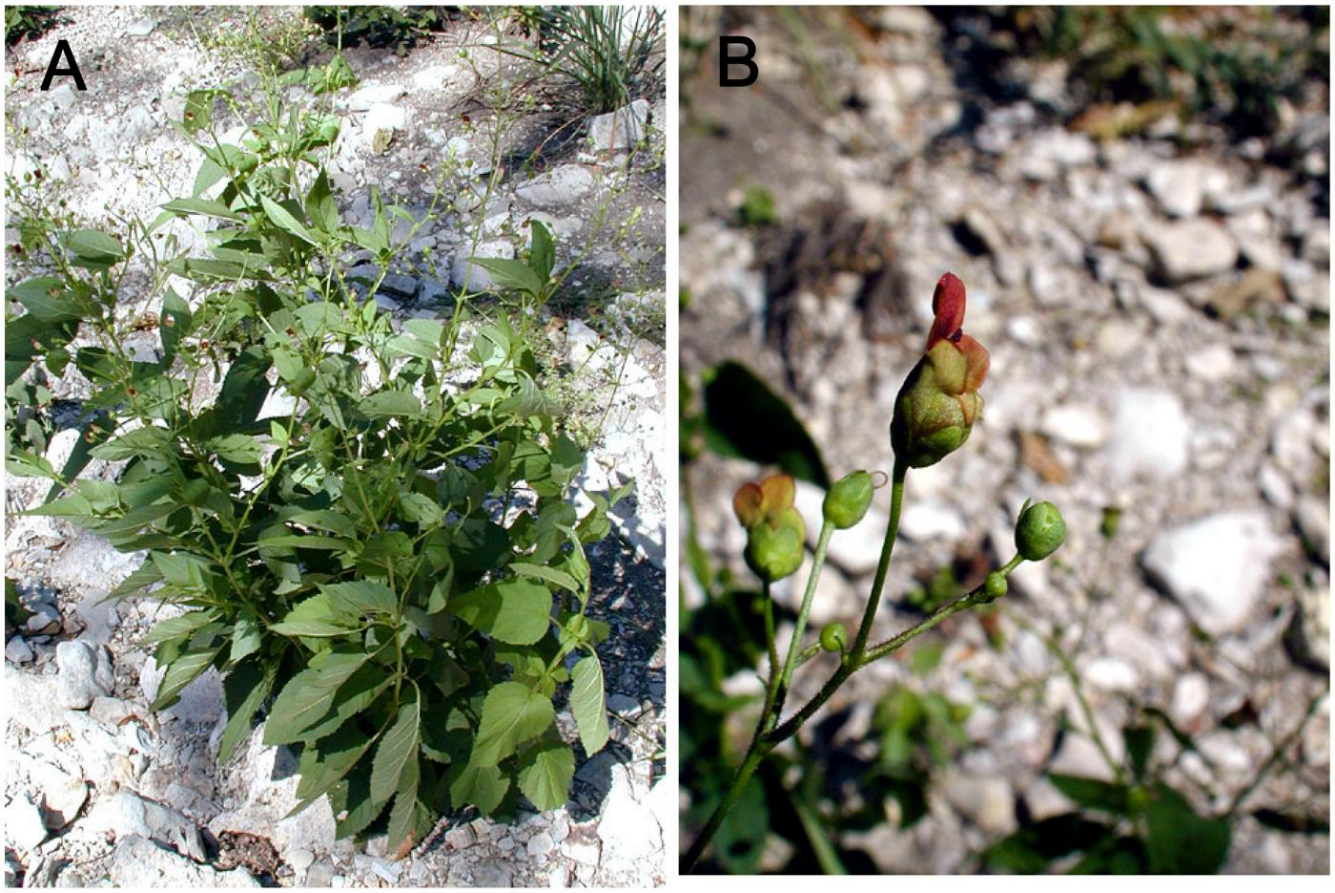
Morphological characteristics of *Scrophularia marilandica:* (A) leaf and (B) flower. These photos were taken by Pan Li from Zhejiang University at Saint Francois, Missouri, United States and we have obtained the permission to include the images in this article. *Scrophularia marilandica* is characterized by opposite leaves, ovate to lanceolate, with serrated margins and a pointed tip. The flowers are arranged in a loose, terminal panicle or thyrse, with small, reddish-brown flowers on slender stalks.

Fresh leaf samples of *S. marilandica* were collected from Moster hollow trail, Saint Francois, Missouri, United States (37.9748 N, 90.5318 W), deposited at Zhejiang Sci-Tech University Herbarium (Voucher No. ZQI00057, Lei Guo, gl200064@163.com) and identified by Ruihong Wang (Figure 1). Genomic DNA was meticulously extracted employing a modified CTAB protocol by using DNA Plantzol reagent (Invitrogen, Carlsbad, CA) following the manufacturer’s protocol (Yu et al. 2019). The constructed libraries were sequenced on the DNBSeq platform using PE100 strategy at China National GenBank (CNGB) in ShenZhen, China. Following data acquisition, raw sequencing reads were quality-filtered and adapter-timmed using Trimmomatic v0.39 (Bolger et al. 2014). The per-base coverage depth across the *S. marilandica* chloroplast genome was analyzed using Bowtie 2 version 2.5.4 (Langmead and Salzberg 2012). Subsequently, we reconstructed the complete cp genome through an iterative de novo assembly process using GetOrganelle (Jin et al. 2020), incorporating read mapping and gap-filling steps to ensure high-quality genome resolution (Du et al. 2025). For cp genome annotation, we used Geneious software, specifically the Geneious Biologics platform from 2023, as accessed on 10 May 2023 from their official website. The genome map was plotted using the online tool Chloroplot (https://irscope.shinyapps.io/Chloroplot/) (Zheng et al. 2020). CPGView was employed to identify both cis-spliced and trans-spliced genes in the genome (Liu et al. 2012).

Eventually, the complete chloroplast sequence of *S. marilandica* was successfully deposited into the NCBI GenBank database (accession number: PQ679323). Phylogenetic reconstruction of *S. marilandica* based on whole cp genome data was performed with 11 closely related species from Scrophulariaceae family downloaded from NCBI GenBank database, taking *Buddleja brachystachya* and *Buddleja officinalis* as the outgroups. The total of 12 cp genome sequences was aligned using MAFFT V7 with default settings. Subsequently, a phylogenetic tree was constructed employing the maximum likelihood method through IQ-TREE V1.6.8 with the optimization model of K3Pu + F + I (Nguyen et al. 2015). MrBayes V3.2.7 was utilized to perform Bayesian inference phylogenetic tree construction under the best model of TPM1uf + I + G (Ronquist and Huelsenbeck 2003).

## Results

**Figure 2.**
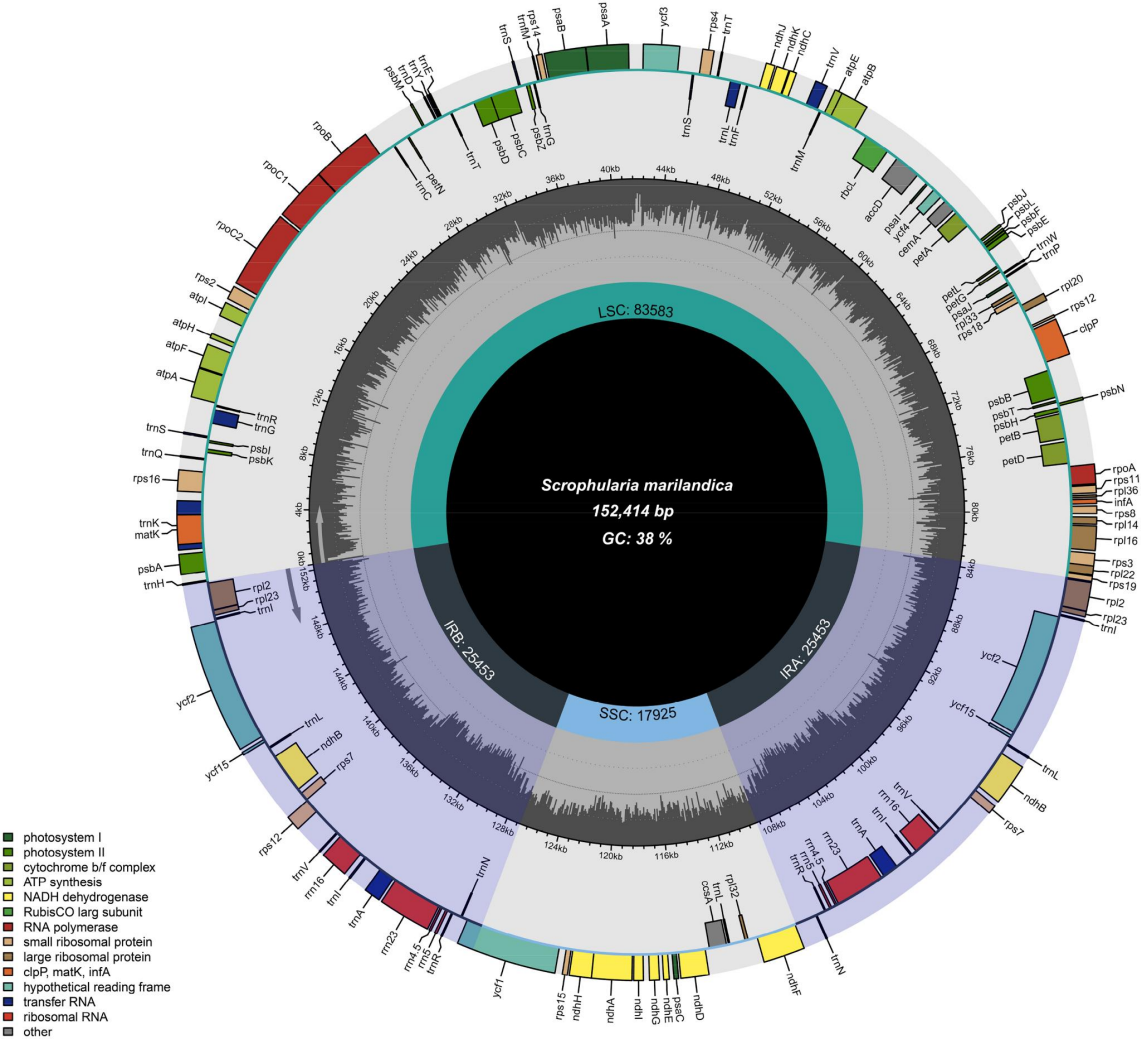
Chloroplast genome map of *Scrophularia marilandica*. Genes are depicted as differently sized and colored boxes on the outermost circle, with inner and outer boxes representing genes transcribed in clockwise and counter-clockwise directions. The middle circle illustrates changes in GC content at different positions, while the inner circle highlights the regions and lengths indicated by the tetrad structure (LSC, SSC, IRa, and IRb) in different colors.

The complete cp genome of *S. marilandica* was 152,414 bp long with an average sequencing depth of 7053x and an overall GC content of 38.0% (Figure S1). The assembled cp genome exhibited a typical quadripartite structure, consisting of a large single-copy region (LSC: 83,583 bp), a small single-copy (SSC: 17,925 bp), and two inverted repeating regions (IRs: 25,453 bp) (Figure 2). The four segments exhibited varying GC content levels, with the IR regions peaking at 43.2%, LSC region in the middle at 36.1%, and the SSC region registering the lowest at 32.2%. In the chloroplast genome of *S. marilandica*, a total of 132 functional genes were identified, comprising 80 protein-coding genes, four ribosomal RNA (rRNA) genes, 30 transfer RNA (tRNA) genes and 18 repeated genes. Of these 114 unique genes, nine protein-coding genes (*petB*, *petD*, *atpF*, *ndhA*, *ndhB*, *rpl16*, *rpl2*, *rps16*, *rpoC1*) and five tRNA genes (*trnA-UGC*, *trnG-GCC*, *trnI-GAU*, *trnL-UAA*, *trnV-UAC*) contained a single intron, while three genes (*clpP, rps12* and *ycf3*) possessed two introns, as illustrated in Supplemental Figure S2. These genes are likely candidates for cis or trans-splicing events during transcription, as further depicted in Supplemental Figures S2 and S3.

**Figure 3.**
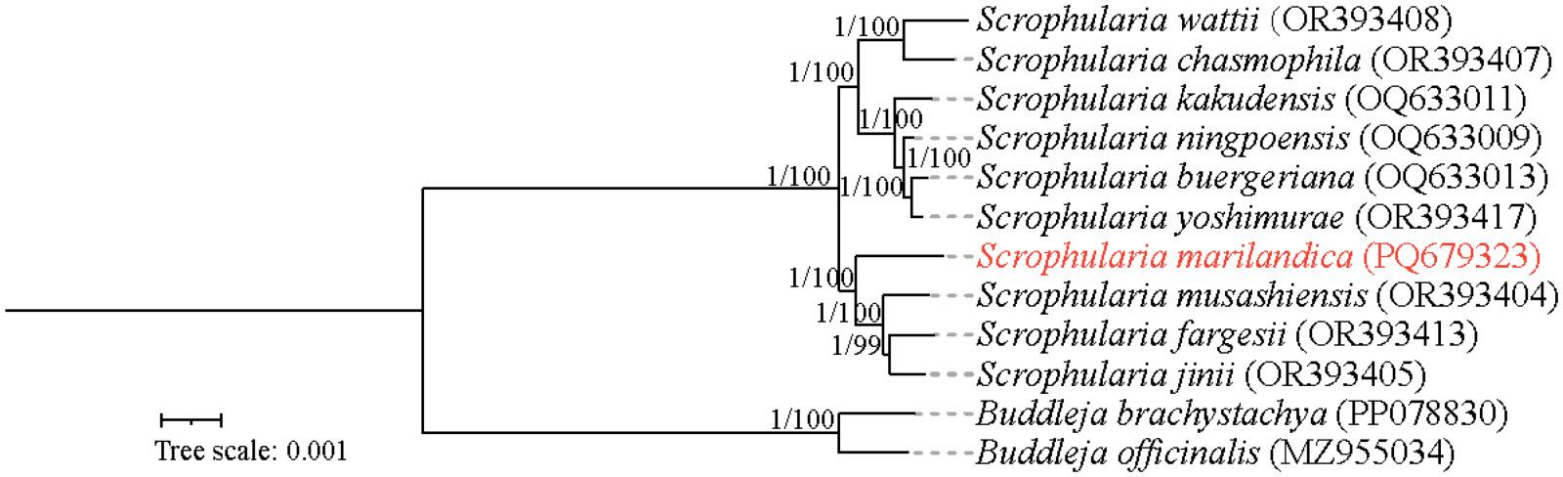
The Phylogenetic tree based on the complete chloroplast genomes of 12 species of Scrophulariaceae, with *Buddleja* as the outgroup. The number values at the nodes represent both the maximum likelihood bootstrap and the Bayesian inference posterior probability. The following details the sequence used to build the phylogenetic tree: the sequences of *Scrophularia wattii* OR393408 (Wang et al. 2024), *Scrophularia chasmophila* OR393407 (Wang et al. 2024), *Scrophularia yoshimurae* OR393417 (Wang et al. 2024), *Scrophularia musashiensis* OR393404 (Wang et al. 2024), *Scrophularia fargesii* OR393413 (Wang et al. 2024), and *Scrophularia jinii* OR393405 (Wang et al. 2024); the sequences of *Scrophularia kakudensis* OQ633011 (Guo et al. 2023), *Scrophularia ningpoensis* OQ633009 (Guo et al. 2023), and *Scrophularia buergeriana* OQ633013 (Guo et al. 2023).

The phylogenetic placement of *S. marilandica*, as a representative of the *Scrophularia* genus, was examined using a dataset comprising 11 species across the Scrophulariaceae family. Bayesian inference (BI) and maximum likelihood (ML) approaches were utilized, yielding congruent topological results, as depicted in Figure 3. The analysis revealed that nearly all the phylogenetic nodes received a 100% confidence level. The outgroup *Buddleja brachystachya* and *Buddleja officinalis* formed a single branch, while the remaining ten species of the *Scrophularia* genus were distinctly separated from the outgroup and formed another large branch. This large branch was then divided into two distinct sister clades. *S. wattii*, *S. chasmophila*, *S. kakudensis*, *S. ningpoensis, S. buergeriana* and *S. yoshimurae* formed one of the groups. *S. musashiensis*, *S. fargesii*, *S. jinii*, and *S. marilandica* studied in this experiment formed another group. The phylogenetic tree indicated that *S. marilandica* has a closer phylogenetic relationship with *S. musashiensis, S. fargesii* and *S. jinii* on the tree.

## Discussion and conclusion

Phylogenetic analysis using complete cp genome sequences of *S. marilandica* and other species of *Scrophularia*, along with the genus *Buddleja* from the Scrophulariaceae family, firmly established *S. marilandica* as a closely related species to *S. musashiensis, S. fargesii* and *S. jinii* on the tree, which is inconsistent with previous phylogenetic tree based on the combined dataset of ITS, *trnQ*-*rps16* and *psbA*-*trnH* (Scheunert and Heubl 2011). This discrepancy may be due to insufficient sampling of North American species in the current study and inadequate informative sites in prior research, leading to low resolution in the phylogenetic tree.

The complete cp genome of *S. marilandica* was firstly sequenced and characterized in the study, uncovering its quintessential quadripartite and circular DNA architecture. Our findings illuminated significant structural and sequence divergence aspects, encompassing genome size, gene and intron composition. This foundational understanding of the chloroplast genome of *S. marilandica* is set to enhence its utility in comparative genomics and enrich the dataset for exploring its pharmacological mechanisms.

## Supplementary Material

Supplemental Material

Supplemental Material

Supplemental Material

## Data Availability

The genome sequence data that support the findings of this study are openly available in GenBank of NCBI at (https://www.ncbi.nlm.nih.gov/) under the accession no. PQ679323. The associated BioProject, SRA, and Bio-Sample numbers are PRJNA1209342, SRR31972123, and SAMN46218880 respectively.
