# Supplementary figures and images for: Chloroplast genome characteristics and phylogenetic analysis of Scrophularia marilandica Linnaeus (Scrophulariaceae)
Source: Mitochondrial DNA B Resour. 2025 Sep 3;10(10):914–8. doi: 10.1080/23802359.2025.2555458 (PMC12409920; doi:10.1080/23802359.2025.2555458)

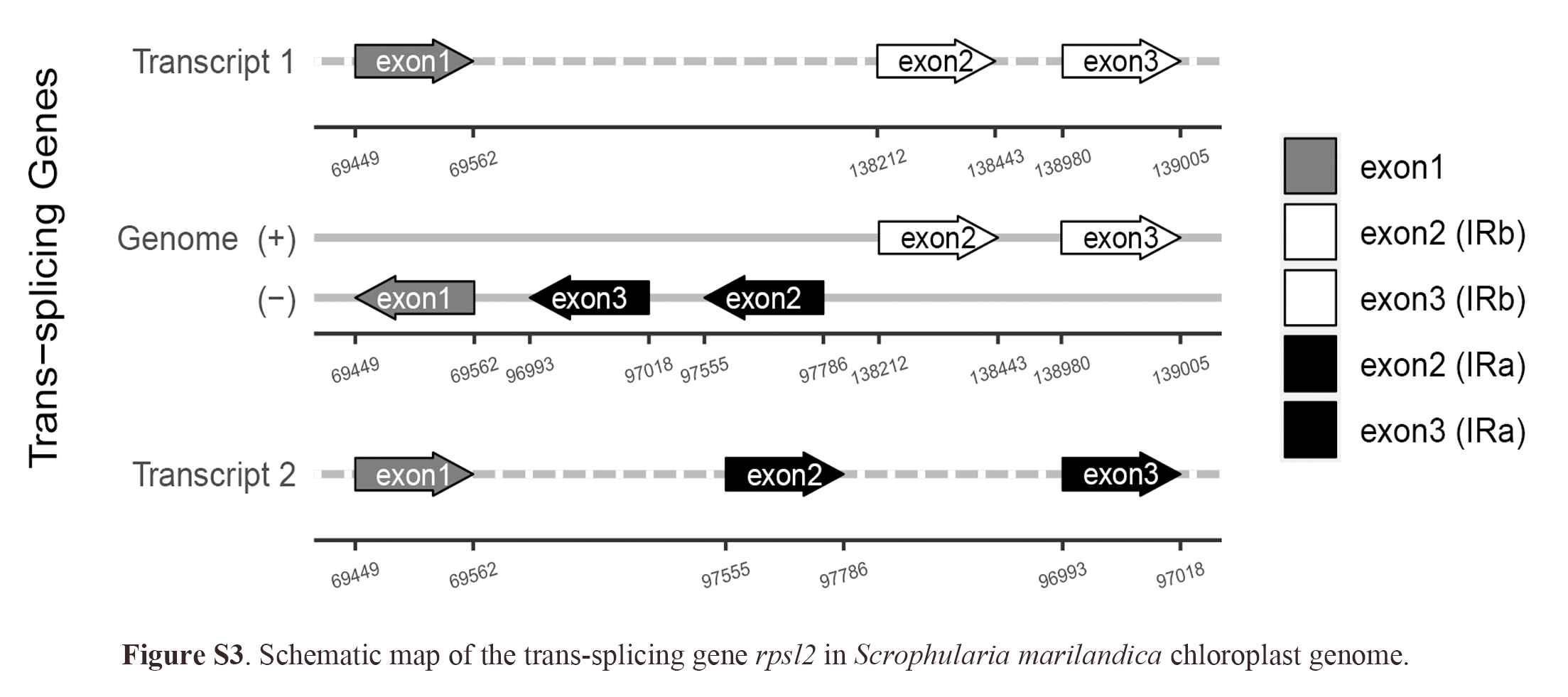

Supplement: Supplemental Material [file TMDN_A_2555458_SM4485.png]

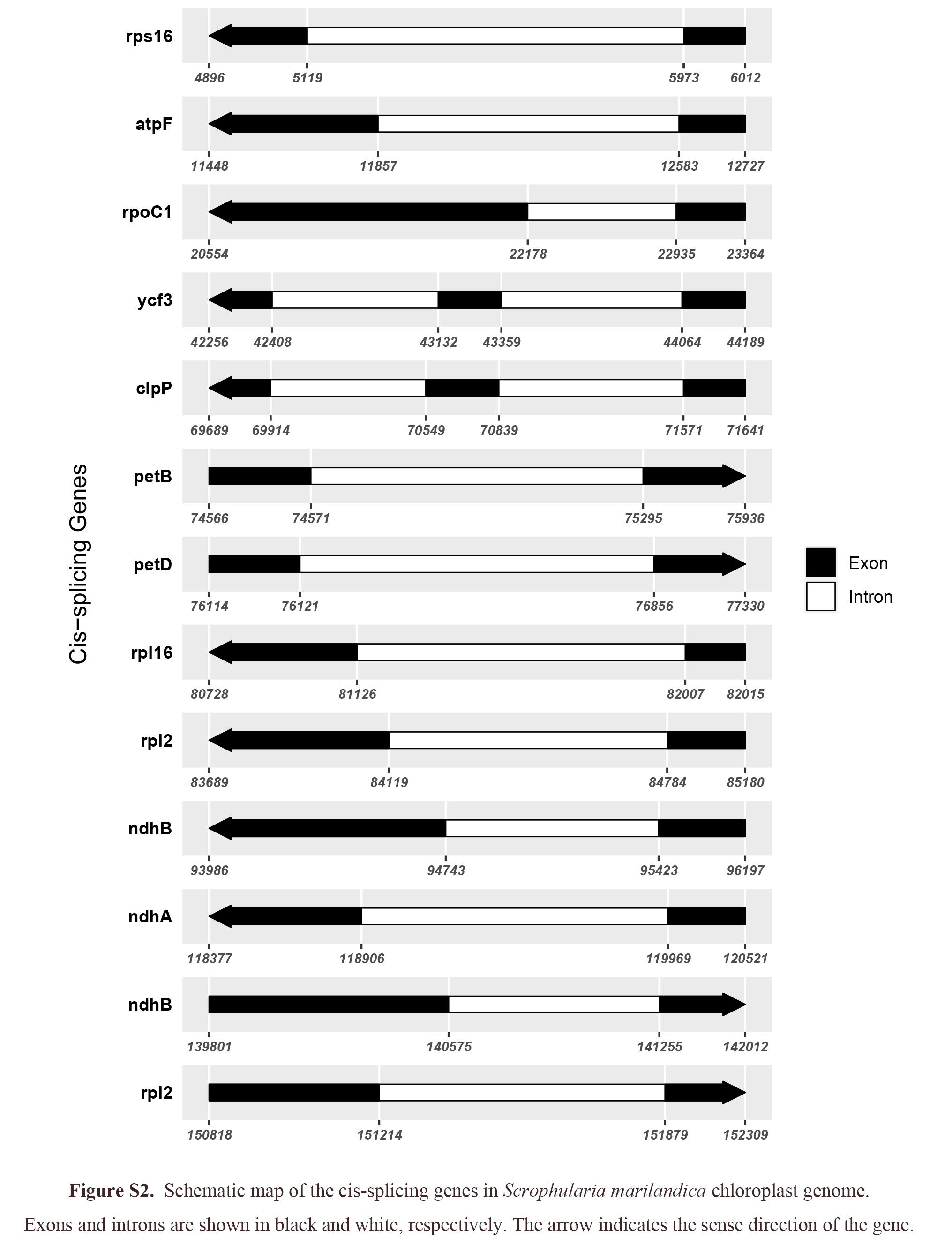

Supplement: Supplemental Material [file TMDN_A_2555458_SM4478.tif]

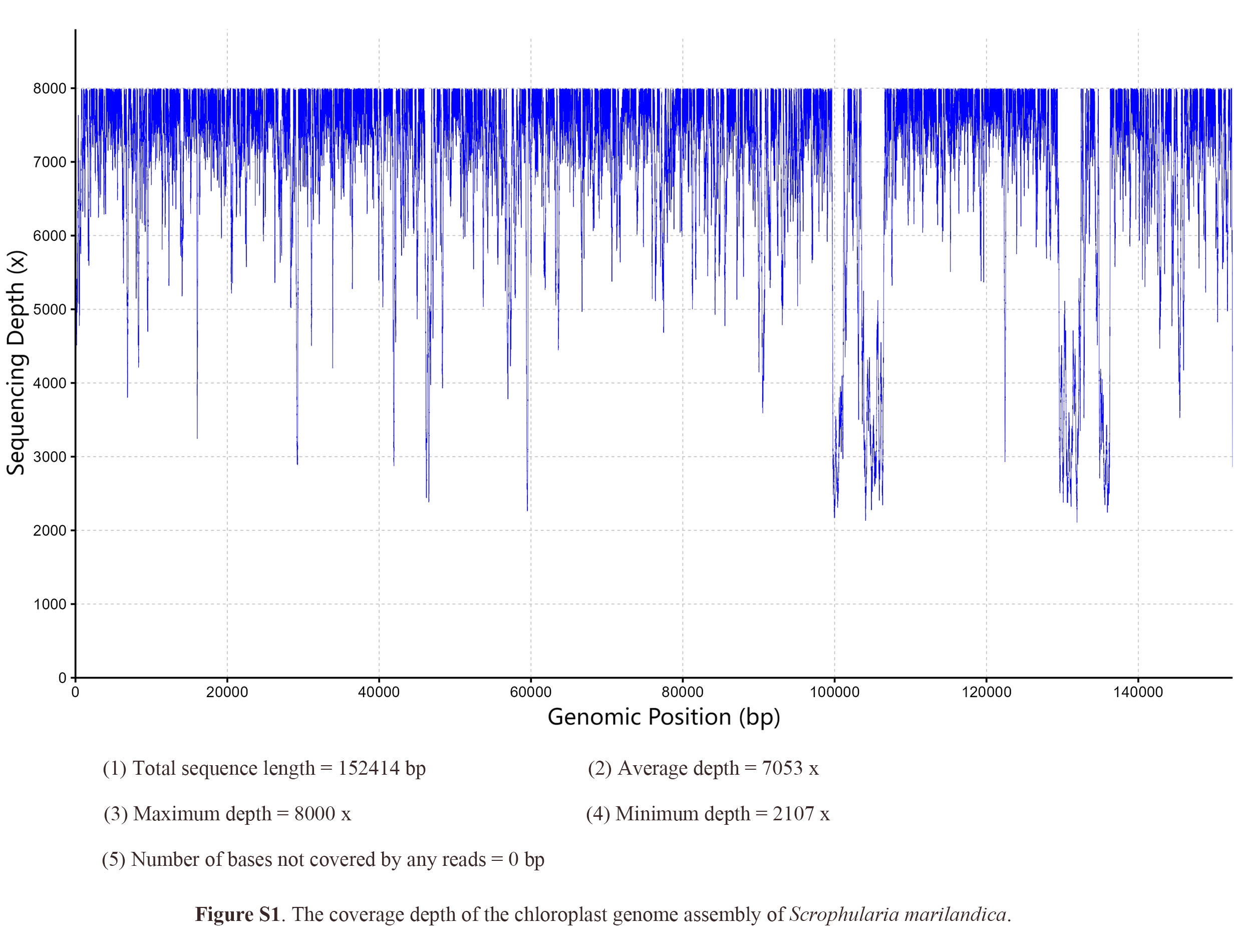

Supplement: Supplemental Material [file TMDN_A_2555458_SM4477.tif]
